# Functional dominance rather than taxonomic diversity and functional diversity mainly affects community aboveground biomass in the Inner Mongolia grassland

**DOI:** 10.1002/ece3.2778

**Published:** 2017-02-09

**Authors:** Qing Zhang, Alexander Buyantuev, Frank Yonghong Li, Lin Jiang, Jianming Niu, Yong Ding, Sarula Kang, Wenjing Ma

**Affiliations:** ^1^ School of Ecology and EnvironmentInner Mongolia UniversityHohhotChina; ^2^ Department of Geography and PlanningUniversity at AlbanyState University of New YorkAlbanyNYUSA; ^3^ School of BiologyGeorgia Institute of TechnologyAtlantaGAUSA; ^4^ Grassland Research Institute of Chinese Academy of Agricultural SciencesHohhotChina

**Keywords:** complementarity effect, functional diversity, functional dominance, Inner Mongolia grassland, selection effect, taxonomic diversity

## Abstract

The relationship between biodiversity and productivity has been a hot topic in ecology. However, the relative importance of taxonomic diversity and functional characteristics (including functional dominance and functional diversity) in maintaining community productivity and the underlying mechanisms (including selection and complementarity effects) of the relationship between diversity and community productivity have been widely controversial. In this study, 194 sites were surveyed in five grassland types along a precipitation gradient in the Inner Mongolia grassland of China. The relationships between taxonomic diversity (species richness and the Shannon–Weaver index), functional dominance (the community‐weighted mean of four plant traits), functional diversity (Rao's quadratic entropy), and community aboveground biomass were analyzed. The results showed that (1) taxonomic diversity, functional dominance, functional diversity, and community aboveground biomass all increased from low to high precipitation grassland types; (2) there were significant positive linear relationships between taxonomic diversity, functional dominance, functional diversity, and community aboveground biomass; (3) the effect of functional characteristics on community aboveground biomass is greater than that of taxonomic diversity; and (4) community aboveground biomass depends on the community‐weighted mean plant height, which explained 57.1% of the variation in the community aboveground biomass. Our results suggested that functional dominance rather than taxonomic diversity and functional diversity mainly determines community productivity and that the selection effect plays a dominant role in maintaining the relationship between biodiversity and community productivity in the Inner Mongolia grassland.

## 1 Introduction

With the accelerating loss of biodiversity, it is urgent to evaluate its consequences for ecosystem functions, such as primary productivity (Hooper et al., 2005; Majekova, de Bello, Dolezal, & Leps, 2014; Paquette, Joly, & Messier, 2015; Venail et al., 2015). Unsurprisingly, the relationship between biodiversity and productivity has been a hot topic in ecology (Grace et al., 2016; Hooper et al., 2005). A number of different forms of such relationships have been found, including positive, negative, and unimodal. Furthermore, these relationships vary at different ecological and geographical scales, with ecosystem type, and with the statistical methods used (Fraser et al., 2015; Grace et al., 2007, 2016; Wang et al., 2011).

While much has been learned about biodiversity–productivity relationships, two controversial issues still require attention. The first is the relative importance of different kinds of biodiversity (including taxonomic diversity, functional diversity, and functional dominance) in maintaining community productivity. These kinds of diversity are each known to determine community productivity, but their relative importance remains highly controversial (Fu et al., 2014; Tobner et al., 2016). Most previous studies have focused mainly on taxonomic diversity (e.g., species richness) as a measure of biodiversity, which has been demonstrated to be important for productivity (Bai, Han, Wu, Chen, & Li, 2004; Venail et al., 2015; Wang et al., 2011). The role of plant functional characteristics has received a great deal of attention in the past 10 years (Cornelissen et al., 2003; Flynn, Mirotchnick, Jain, Palmer, & Naeem, 2011; Fry, Power, & Manning, 2014; Jain et al., 2014; Liu et al., 2015). Many studies have found functional characteristics to be a key component that often explains productivity better than taxonomic diversity (Finegan et al., 2015; Fu et al., 2014; Siebenkas & Roscher, 2016). Two dimensions of functional characteristics—functional dominance (or functional identity) and functional diversity—are also believed to influence productivity (Chanteloup & Bonis, 2013; Fu et al., 2014; Tobner et al., 2016). The former reflects the effects of the functional characteristics of the dominant species on community productivity (Chanteloup & Bonis, 2013; Tobner et al., 2016). The latter mainly refers to the effect of the variation in the functional characteristics of all species within a community on community productivity (Cavanaugh et al., 2014; Fu et al., 2014). When comparing functional dominance and functional diversity, recent studies have demonstrated that functional dominance is more important in community productivity than functional diversity (Li et al., 2015; Mokany, Ash, & Roxburgh, 2008; Tobner et al., 2016). However, some studies have found that functional diversity plays a more important role in community productivity than functional dominance (Finegan et al., 2015; Siebenkäs, Schumacher, & Roscher, 2016).

The second issue is related to the underlying mechanisms of the relationship between diversity and productivity, which include selection and complementarity effects. The selection effect predicts that a more diverse community is more likely to contain species with higher productivity (dominant species), which then determines ecosystem productivity (Grime, 1998; Roscher et al., 2012). The complementarity effect occurs when a more diverse community may exhibit higher variance in functional traits, which increases the optimal use of resources and enhances ecosystem productivity (Cavanaugh et al., 2014; Tilman, Lehman, & Thomson, 1997). A meta‐analysis conducted by Cardinale et al. (2011) demonstrated that selection and complementarity effects contribute to the net diversity effect approximately equally. Meanwhile, these two effects are not mutually exclusive and may operate simultaneously. However, the relative importance of selection and complementarity effects in maintaining the relationship between biodiversity and productivity varies with different ecosystem types, research scales and successional gradients (Cardinale et al., 2011; Cavanaugh et al., 2014; Finegan et al., 2015; Siebenkäs et al., 2016).

**Figure 1:**
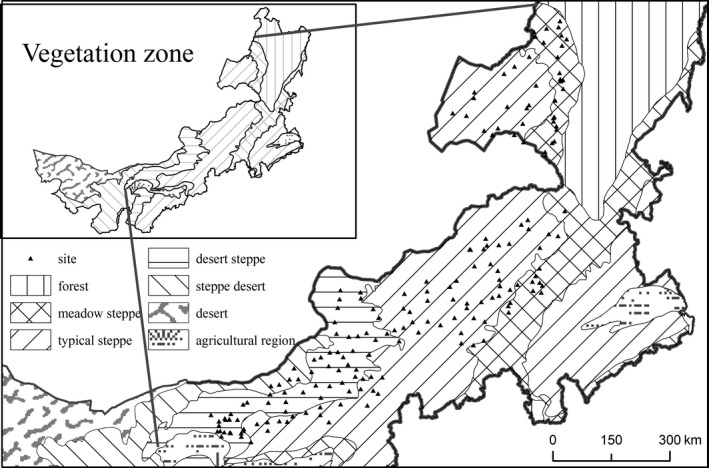
Study area and field sampling sites. The five vegetation zones are forest, meadow steppe (mature herb synusia, dominated by mesophyte plants), typical steppe (mature herb synusia, dominated by xerophyte plants), desert steppe (dominant herb synusia, with developed shrub synusia), steppe desert (dominant shrub synusia, with developed herb synusia), and desert. The solid triangle indicates the field site

The Inner Mongolia grassland is part of the Eurasian steppe, which extends over 8,000 km from northeastern China, Mongolia, Russia and Ukraine to Hungary (Coupland, 1993). Along the east–west precipitation gradient in the area, three vegetation zones are formed, including meadow steppe (mature herb synusia dominated by mesophytes), typical steppe (mature herb synusia dominated by xerophytes), and desert steppe (dominant herb synusia with developed shrub synusia) (Figure 1). Thus, in this study, we aimed to explore the relative importance of taxonomic diversity, functional dominance, and functional diversity on community productivity along the precipitation gradient in the Inner Mongolia grassland. We conducted a field survey of 194 sites in five grassland types along the precipitation gradient in the Inner Mongolia grassland of China. First, we test how taxonomic diversity, functional dominance, functional diversity, and community productivity change along the gradient. Second, we assess the relationships among taxonomic diversity, functional dominance, functional diversity, and community productivity. Third, we explore the relative importance of taxonomic diversity and functional characteristics on community aboveground biomass. Finally, we examine whether the effects of functional characteristics on community productivity are largely dependent on specific traits.

## 2 Materials and Methods

### 2.1 Study area

This study was conducted across the Inner Mongolia grasslands in northern China. The region stretches from 41.31°N to 50.78°N in latitude and from 108.16°E to 120.39°E in longitude with the elevation ranging from 532 to 1,725 m above sea level (Figure 1). Typical landforms in this region include gently rolling plains, tablelands, and hills. The mean annual temperature ranges from −3.0 to 6.7°C, and the mean annual precipitation varies from approximately 150 to 450 mm, decreasing from east to west (Wu, Zhang, Li, & Liang, 2015; Zhang et al., 2014). The precipitation gradient drives the distribution of plant–soil complexes from the meadow steppe over chernozems in the east to the typical steppe over chestnut soil and desert steppe over calcic brown soils in the west (Figure 1).

### 2.2 Data collection

Our vegetation survey was conducted in 2012 during the peak of aboveground biomass, which occurs from late July to mid‐August. Five grassland zones increasing in mean annual precipitation were investigated (Table 1), including *Stipa klemenzii* desert steppe, *S. breviflora* desert steppe, *S. krylovii* typical steppe, *S. grandis* typical steppe, and *S. baicalensis* meadow steppe. A total of 194 sites spaced 30–40 km were surveyed. The number of sites per zone was proportional to the area occupied by each steppe zone (Bai et al., 2007) (Figure 1). To exclude the effects of grazing on diversity and productivity, the sites were selected based on the availability of individually fenced exclosure pastures that had experienced no grazing for at least 3 years. At each site, a 10 × 10 m area was chosen in the center of a single fenced exclosure pasture, and three 1 × 1 m quadrats were then randomly placed within this area. The results from the three quadrats were averaged for further analyses. In each quadrat, we recorded the species and harvested and weighed the aboveground parts of the plants. The dry weight was obtained by oven‐drying at 60°C for 24 hr to achieve a constant weight. The average aboveground biomass (AGB) from the three quadrats was used as a measure of the community productivity at each site (Bai et al., 2007).

**Table 1:** Abiotic and biotic characteristics of five grassland types in the Inner Mongolia grassland

Grassland types	*Stipa klemenzii* desert steppe	*S. breviflora* desert steppe	*S. krylovii* typical steppe	*S. grandis* typical steppe	*S. baicalensis* meadow steppe	Inner Mongolia grassland
No. of plots	40	39	35	44	36	194
MAP (mm)	175.11 ± 13.82	225.29 ± 15.29	273.14 ± 21.14	326.25 ± 26.25	372.5 ± 19.65	273.88 ± 72.06
MAT (°C)	3.19 ± 1.25	2.64 ± 1.46	1.01 ± 0.51	−0.73 ± 0.67	−1.00 ± 0.52	1.02 ± 0.92
AGB (g)	76.47 ± 31.39	101.31 ± 35.76	139.51 ± 59.01	187.34 ± 48.18	203.82 ± 36.21	141.62 ± 72.23
S	10.90 ± 2.91	13.67 ± 4.15	15.37 ± 3.59	22.70 ± 10.77	25.19 ± 9.06	17.59 ± 8.84
*H′*	0.95 ± 0.53	1.10 ± 0.46	1.18 ± 0.42	1.64 ± 0.62	1.75 ± 0.50	1.33 ± 0.60
CWM_H_ (cm)	15.31 ± 4.87	22.17 ± 1.37	40.41 ± 19.98	47.34 ± 14.46	43.20 ± 12.80	33.66 ± 18.78
CWM_LA_ (cm^2^)	0.64 ± 0.19	0.68 ± 0.27	1.35 ± .0.96	3.32 ± 1.59	4.42 ± 2.29	2.09 ± 1.99
CWM_LDM_ (g)	0.012 ± 0.004	0.04 ± 0.02	0.05 ± 0.05	0.06 ± 0.04	0.06 ± 0.03	0.04 ± .04
CWM_SLA_ (cm^2^/g)	64.94 ± 22.74	73.73 ± 22.27	81.39 ± 21.55	76.81 ± 19.47	91.89 ± 24.96	77.37 ± 23.63
FD_Q_	0.22 ± 0.12	0.88 ± 0.46	1.96 ± 1.45	3.09 ± 2.35	3.53 ± 2.63	1.93 ± 1.21

MAP, mean annual precipitation; MAT, mean annual temperature; AGB, aboveground biomass; S, species richness; *H′*, Shannon–Weaver index; CWM_H_, community‐weighted mean of height; CWM_LA_, community‐weighted mean of leaf area; CWM_LDM_, community‐weighted mean of leaf dry weight; CWM_SLA_, community‐weighted mean of specific leaf area; FD_Q_, Rao's quadratic entropy.

Species traits were recorded for all healthy and pest‐free plants of all the species found at all sites following Cornelissen et al. (2003). The plant height (H) was measured for 15 individuals of common species (frequency of more than 0.2) and three individuals of occasional species (frequency of less than 0.2). Three intact leaves from each plant were collected, and the leaf area (LA) was measured using a leaf area meter (LI‐3100 Area Meter, LI‐COR, Lincoln, USA). After oven‐drying the leaves at 60°C, the leaf dry weight (LDM) was obtained, and the specific leaf area (SLA) was calculated based on the LA and LDM.

Soil samples were collected from three 30‐cm‐deep cores within each quadrat. The three cores were later mixed in the laboratory. The total nitrogen was determined using the selenium‐cupric sulfate (CuSO_4_)‐potassium sulfate (K_2_SO_4_)‐heating digestion method. The total phosphorus was measured by the alkali fusion‐Mo‐Sb colorimetric method. The available nitrogen (${\text{NH}}_{4}^{+}$ and ${\text{NO}}_{3}^{-}$) was measured by the Kjeldahl nitrogen determination method. The available phosphorus was measured by the sodium bicarbonate (NaHCO_3_) leaching‐Mo‐Sb colorimetric method. The organic carbon was measured by the potassium dichromate (K_2_Cr_2_O_7_) heating oxidation method (Qiao, 2012).

The mean annual precipitation and temperature as well as the precipitation and temperature of the wettest and driest quarters were obtained at 30 arc‐second (~1 km^2^) resolution from the WorldClim database (www.worldclim.org) (Hijmans, Cameron, Parra, Jones, & Jarvis, 2005).

### 2.3 Diversity and trait metrics

The taxonomic diversity was expressed as the species richness (S) and the Shannon–Weaver index (*H′*) as shown below:

where *S*_*i*_ is the number of species in each quadrat, and

$$S=\frac{1}{3}\sum_{i=1}^{3}S_{i}$$

$$H^{\prime }=-\sum_{i=1}^{S}p_{i}\times \log_{2}\left(p_{i}\right)$$

where *p*_*i*_ is the importance value of species *i* in the community, calculated as the average of the proportion of the dry weight of species *i* out of the total dry weight in the three quadrats, and *S* is the number of species.

The functional dominance was expressed by the CWM (Fu et al., 2014; Roscher et al., 2012):

$$\text{CWM}=\sum_{i=1}^{S}p_{i}\times {\text{trait}}_{i}$$

where *S* is the total number of species, *p*_*i*_ is the relative abundance of species *i,* and “trait_*i*_” is the trait value of species *i*. When ecosystem function refers to productivity, it is reasonable to use the proportion of biomass as an indicator of the relative abundance (Garnier et al., 2004). Therefore, *p*_*i*_ is calculated in the same way as for the Shannon–Weaver index. We considered several traits, including plant height (H), leaf area (LA), leaf dry weight (LDM), and specific leaf area (SLA).

Functional diversity was expressed as FD_Q_ (Fu et al., 2014; Roscher et al., 2012).

$${\text{FD}}_{Q}=\sum_{i=1}^{S}\sum_{j=1}^{S}d_{ij}p_{i}p_{j}$$

$$d_{ij}=\sum_{k=1}^{n}\sum_{l=1}^{n}w_{kl}\left(X_{ik}-X_{jk}\right)\left(X_{il}-X_{jl}\right)X_{il}-X_{jl}$$

where *S* is the number of species; *p*_*i*_ and *p*_*j*_ are the relative abundances of species *i* and *j*, respectively, calculated the same way as for the CWM; *d*_*ij*_ (0 ≤ *d*_*ij*_ ≤ 1) is the Mahalanobis distance, reflecting the difference in trait characteristics between species *i* and *j* (Botta‐Dukat, 2005) (*d*_*ij*_ = 0 when the trait values are identical and *d*_*ij*_ = 1 when they are completely different); *w*_*kl*_ represents the elements of the inverse of the variance–covariance matrix of the traits; and *X*_*ik*_ is the value of trait *k* for species *i*.

### 2.4 Statistical analysis

To reveal the changes in taxonomic diversity, functional dominance, functional diversity, and community aboveground biomass along the precipitation gradient, we calculated the means of the related indices (including species richness, the Shannon–Weaver index, CWM_H_, CWM_LA_, CWM_LDM_, CWM_SLA_, FD_Q_, and AGB) for the five grassland types.

To assess the relationship between diversity and productivity, we performed ordinary least squares regression to examine the relationships between community aboveground biomass and species richness, the Shannon–Weaver index, CWM_H_, CWM_LA_, CWM_LDM_, CWM_SLA_, and FD_Q_.

To explore the relative importance of taxonomic diversity and functional characteristics on community aboveground biomass, the explanatory power of different groups of predictor variables (including environmental factors, taxonomic diversity, and functional characteristics) in the variation in community aboveground biomass of 194 sites was evaluated using a nested multiple regression model analysis (Fu et al., 2014; Roscher et al., 2012): (1) environmental factors, including the 11 precipitation, temperature and soil variables that were introduced in the Methods section; (2) species diversity, including species richness and the Shannon–Weaver index; (3) functional characteristics, including CWM_H_, CWM_LA_, CWM_LDM_, CWM_SLA_, and FD_Q_; and (4) the final model including the remaining significant variables for each predictor group. First, we fitted linear regression models with each single predictor variable to evaluate their significance in explaining the variation in community aboveground biomass. Second, models initially fitted with all significant variables per predictor group as fixed effects were simplified through stepwise exclusion of nonsignificant variables. Third, the remaining candidate variables per predictor group were entered into a combined model. The combined model was successively reduced by eliminating nonsignificant interaction terms first and nonsignificant main effects afterward.

To examine whether the effects of the functional characteristics on productivity are largely dependent on specific traits, multiple stepwise regression was used to generate predictive models for AGB based on the functional diversity and functional dominance (Chanteloup & Bonis, 2013). In the predictive models, only variables with *p* < .05 were selected using backward stepwise selection. The adjusted *R* ^2^ for each variable was used to assess its explanatory power in predicting AGB (Wang, Fang, Tang, & Lin, 2012). This is helpful in determining key functional traits affecting community aboveground biomass.

Indices of taxonomic diversity, functional dominance, and functional diversity were calculated using the FDiversity software (Casanoves, Pla, Di Rienzo, & Diaz, 2011). Ordinary least squares regression and multiple stepwise regression analyses were conducted using SPSS 17.0 software.

## 3 Results

### 3.1 Taxonomic diversity, functional dominance, functional diversity, and aboveground biomass along the precipitation gradient

Species richness, the Shannon–Weaver index, the CWM of the four functional traits (height, leaf area, leaf dry weight, and specific leaf area), Rao's quadratic entropy, and aboveground biomass all increased from the grassland type with the lowest precipitation to the grassland type with the highest precipitation (Table 1).

### 3.2 The relationship between taxonomic diversity, functional dominance, functional diversity, and community aboveground biomass

**Figure 2:**
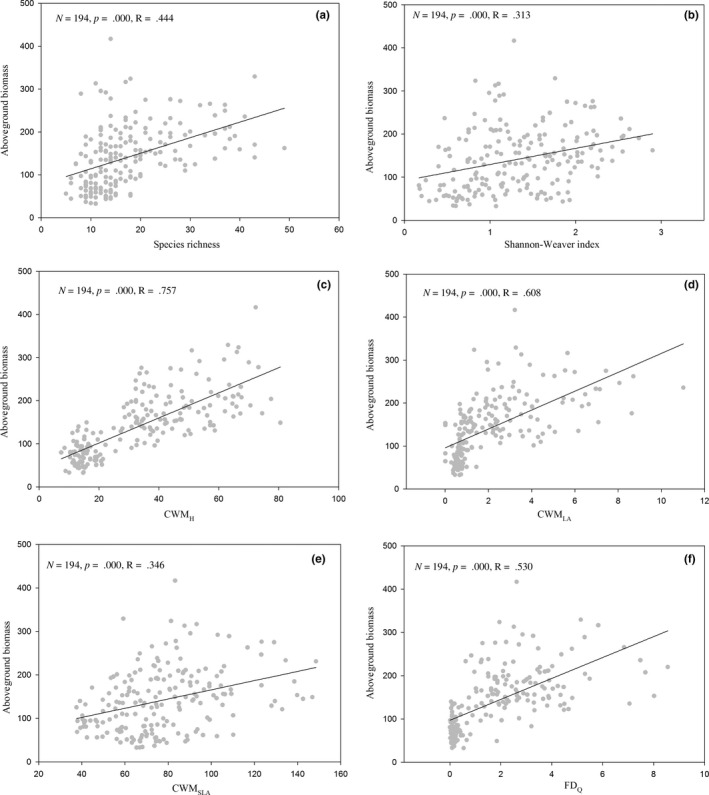
The relationships between taxonomic diversity, functional dominance, functional diversity, and community aboveground biomass in the Inner Mongolia grassland. Notes: (a) species richness, (b) Shannon–Weaver index, (c) community‐weighted mean of height, (d) community‐weighted mean of leaf area, (e) community‐weighted mean of specific leaf area, (f) Rao's quadratic entropy. CWM_H_, community‐weighted mean of height; CWM_LA_, community‐weighted mean of leaf area; CWM_SLA_, community‐weighted mean of specific leaf area; FD_Q_, Rao's quadratic entropy

In the Inner Mongolia grassland, significant positive correlations were found between community aboveground biomass and species richness (Figure 2a), the Shannon–Weaver index (Figure 2b), CWM_H_ (Figure 2c), CWM_LA_ (Figure 2d), CWM_SLA_ (Figure 2e), and FD_Q_ (Figure 2f). However, there was no significant correlation between community aboveground biomass and CWM_LDM_.

### 3.3 The relative importance of environmental factors, taxonomic diversity, and functional characteristics in determining community aboveground biomass

Functional characteristics were more important in determining community aboveground biomass than taxonomic diversity in the Inner Mongolia grassland and the five grassland types individually (Table 2). Meanwhile, the results show that the explanatory power of the environmental factors decreases along the gradient of increasing precipitation (Table 2).

**Table 2:** Explanatory power of different groups of predictor variables (environmental factors, taxonomic diversity, and functional characteristics) in the variation in community productivity

Predictor variables	*S. klemenzii* desert steppe	*S. breviflora* desert steppe	*S. krylovii* typical steppe	*S. grandis* typical steppe	*S. baicalensis* meadow steppe	Inner Mongolia grassland
Environmental factors	0.626	0.538	0.479	0.169	0.144	0.570
Taxonomic diversity	0.029	0.224	0.031	0.013	0.109	0.198
Functional characteristics	0.527	0.674	0.503	0.437	0.407	0.679
Final model	0.700	0.822	0.738	0.477	0.507	0.754

### 3.4 Explanatory power of different functional characteristics in community aboveground biomass

In the multiple stepwise regression, both CWM and FD_Q_ were retained in the model for the grassland with the lowest precipitation, the *S. klemenzii* desert steppe (Table 3). For the other steppe types, only the CWMs of the different traits were retained in the regression models. This indicates the important role of plant traits in community aboveground biomass in the Inner Mongolia grassland. The relative importance of different plant traits shifted from leaf area and specific leaf area being the main predictors of community productivity in the *S. klemenzii* desert steppe, which has the lowest precipitation, to plant height being the main predictor in the steppe zones with higher precipitation. Overall, height was the crucial functional trait, explaining 57.1% of the variation in community productivity in the Inner Mongolia grasslands.

**Table 3:** Multiple stepwise regression analysis of predictor variables of selection and complementarity effects in maintaining community aboveground biomass. These variables are standardized with min‐max normalization methods. The final model included the remaining significant variables (*p* < .05). The adjusted *R* ^2^ of each variable in the predictive models was used to assess the predictive power for aboveground biomass

	Predictor variables	Adjusted *R* ^*2*^	β	Significance
*S. klemenzii* desert steppe	CWM_LA_	0.176	.160	.004
	CWM_SLA_	0.320	.640	.000
	FD_Q_	0.390	.826	.000
	Intercept	99.455		
		AGP = 99.455 + 26.078CWM_LA_ − 0.883CWM_SLA_ + 79.701FD_Q_		
*S. breviflora* desert steppe	CWM_H_	0.687	.834	.000
	Intercept	26.257		
		AGP = 26.257 + 3.385CWM_H_		
S. *krylovii* typical steppe	CWM_H_	0.406	.488	.000
	CWM_LA_	0.507	.372	.000
	Intercept	87.587		
		AGP = 87.587 + 1.487CWM_H_ + 23.466CWM_LA_ − 3036.735CWM_SLW_		
*S. grandis* typical steppe	CWM_H_	0.225	.509	.001
	CWM_SLA_	0.326	.208	.000
	CWM_LA_	0.406	.353	.000
	Intercept	32.046		
		AGP = 32.046 + 1.695CWM_H_ + 0.515CWM_SLA_ + 10.683CWM_LA_		
*S. baicalensis* meadow steppe	CWM_H_	0.277	.546	.001
	Intercept	76.404		
		AGP = 76.404 + 2.949CWM_*H*_		
Inner Mongolia grassland	CWM_H_	0.571	.608	.000
	CWM_LA_	0.689	.349	.000
	Intercept	36.589		
		AGP = 36.589 + 2.338CWM_H_ + 1.2614CWM_LA_		

CWM_H_, community‐weighted mean of height; CWM_LA_, community‐weighted mean of leaf area; CWM_SLA_, community‐weighted mean of specific leaf area; FD_Q_, Rao's quadratic entropy.

## 4 Discussion

### 4.1 Positive linear relationships between taxonomic diversity, functional characteristics, and community productivity, and the explanatory power of functional characteristics is stronger than that of taxonomic diversity

Research on the relationship between biodiversity and productivity can be traced back to at least two centuries ago (Diaz & Cabido, 1997). Unraveling the relationships between biodiversity and productivity, including positive, negative, unimodal, or even nonsignificant patterns, remains a primary focus in ecological research (Fraser et al., 2015; Grace et al., 2016; Hooper et al., 2005; Mittelbach et al., 2001). Many studies have demonstrated positive diversity–productivity relationships, at least for grasslands (Bai et al., 2004; Cadotte, Dinnage, & Tilman, 2012; Fu et al., 2014; Grace et al., 2016). Our study also found that both taxonomic diversity (species richness and the Shannon–Weaver index) and functional characteristics (CWM_H_, CWM_LA_, CWM_SLA_, and FD_Q_) are positively correlated with the community aboveground biomass in the Inner Mongolia grassland (Figure 2). Grace et al. (2016) summarized four primary competing theories for the relationships between diversity and productivity as follows: (1) richness and productivity increase together with increasing resources and environmental favorability until limits to coexistence are reached at high productivity and richness declines, producing a hump‐shaped relationship; (2) richness promotes productivity, leading to a positive relationship; (3) richness and productivity increase together because climatic gradients in productivity lead to increased regional species pools, creating a positive relationship but from a separate mechanism; (4) the richness–productivity relationship has an inconsistent form because the mechanisms controlling them vary in their scale dependence and relative importance. We supposed that because of increasing resources along the precipitation gradient, diversity and community productivity should increase together (Table 1), creating a positive relationship in the Inner Mongolia grassland. However, for some individual grassland types, the effects of environmental factors are much weaker than in the entire Inner Mongolia grassland, such as the *S. grandis* typical steppe and the *S. baicalensis* meadow steppe (Table 2), and diversity is the essential factor improving community productivity. We suspected that a negative relationship between taxonomic diversity and community productivity may be found when nutrient availability exceeds a limit. Some nitrogen or phosphorus addition experiments have shown this result (Bernhardt‐Römermann, Römermann, Sperlich, & Schmidt, 2011; Dickson, Mittelbach, Reynolds, & Gross, 2014; Li et al., 2015). Considering the lack of nutrients (Wu et al., 2015), such negative relationships are almost nonexistent in the natural Inner Mongolia grassland.

Some recent studies have found that functional characteristics (including functional dominance or functional diversity) are much more closely related to community productivity and are more important for maintaining productivity than species diversity (Cavanaugh et al., 2014; Flynn et al., 2011; Jain et al., 2014; Roscher et al., 2012; Tobner et al., 2016). Our study of natural grasslands provides new evidence to support this. Our findings show that the contributions of functional characteristics to community productivity are greater than that of taxonomic diversity both in the five grassland types and in the Inner Mongolia grassland as a whole (Table 2). This is because taxonomic diversity does not consider the differences between the functional characteristics of species (Leps, de Bello, Lavorel, & Berman, 2006). Due to the limitations of species distributions, different species in various regions or even populations of the same species located in different regions exhibit varying functional traits (Diaz & Cabido, 2001; Leps et al., 2006). Therefore, communities with the same species richness may exhibit great functional differences due to various species traits (Leps et al., 2006; Roscher et al., 2012). Compared with taxonomic diversity, functional characteristics better reflect the differences in species functional traits, which in turn explains the variation in community productivity (Fu et al., 2014; Li et al., 2015; Tobner et al., 2016). Overall, our findings suggest that increases in productivity are increased both by taxonomic diversity and functional characteristics and that the determinant is functional characteristics, not species number (Diaz & Cabido, 2001; Fu et al., 2014). This demonstrates that taxonomic diversity and functional diversity are two different components of biological diversity and that they are not exactly the same as the alternative (Bu, Zang, & Ding, 2014; Sasaki et al., 2009).

### 4.2 Functional dominance is the best predictor of variability in community aboveground biomass, and the selection effect may be the main mechanism for the diversity effect

Although many studies have demonstrated the importance of functional characteristics in community productivity (Diaz & Cabido, 2001; Wacker, Baudois, Eichenberger‐Glinz, & Schmid, 2009), knowledge regarding the relative roles of functional dominance and functional diversity in explaining the variation in community productivity is still limited (Cavanaugh et al., 2014; Chanteloup & Bonis, 2013; Tobner et al., 2016). Functional dominance is always reflected by the community‐weighted means of different traits. This method identifies the dominant trait values in a community and serves as a predicator of the selection effect (Fu et al., 2014; Li et al., 2015; Roscher et al., 2012). The FD_Q_ evaluates functional diversity by quantifying the differences in functional traits between plant species within a community (Mouchet, Villeger, Mason, & Mouillot, 2010). This method was derived based on the hypothesis that with an increase in the differences in functional traits, the number of the ways in which resources are used also increases (Tilman, 1997). Therefore, FD_Q_ is closely related to the complementarity effect (Chanteloup & Bonis, 2013; Mouchet et al., 2010).

In this study, we applied a multiple stepwise regression to test the relative effects of functional dominance and functional diversity on community aboveground biomass along the precipitation gradient. The results showed that the community‐weighted means of height, leaf area, and specific leaf area were more important in explaining the community aboveground biomass in comparison with the FD_Q_ (Table 3). For example, the community‐weighted means of height and leaf area explained 68.9% of the variation in the community aboveground biomass. Our results supported the selection effect, showing that the functional traits of the dominant species largely contribute to the community aboveground biomass. Our results are consistent with previous studies indicating that functional dominance may be the main driver determining variation in community productivity (Finegan et al., 2015; Fu et al., 2014; Tobner et al., 2016).

However, in contrast to the regression models for the other four grassland types, FD_Q_ was retained in the regression model for the *S. klemenzii* desert steppe zones, and the combination of CWM and FD_Q_ largely promoted the explanatory power of the regression models (Table 3). This result suggests that the selection effect and the complementarity effect are not mutually exclusive in explaining the relationship between biodiversity and community productivity in the extremely dry steppe grassland; they simultaneously operate and explain 39% of the variance in the community aboveground biomass (Cardinale et al., 2011; Fu et al., 2014). We speculate that these results may be an outcome of drought screening. The *S. klemenzii* desert steppe is the driest grassland type of the Inner Mongolia grassland. Such drought screening reduces plant functional trait differences among different species and consequently causes the aggregation of plant functional traits (Schellberg & Pontes, 2012). Thus, the increase in species number could not obviously lead to an increase in CWM. The relative importance of the selection effect would become weak under these conditions. Increasing species numbers allow the occupation of more ecological niches and further lead to the complementary use of resources (Tilman et al., 1997). Therefore, this should increase the FD_Q_ value and enhance the complementarity effect. Meanwhile, it is conceivable that there would be greater complementarity (or competition) belowground than aboveground for limiting resources (water and nutrients) (Chanteloup & Bonis, 2013; Siebenkas & Roscher, 2016). Thus, aboveground biomass and aboveground plant functional traits may be poor predictors for explaining the variation in community productivity, indicating that plant root functional traits and leaf nutrient traits may be more appropriate. We should strengthen the research about plant root functional traits and community belowground productivity. In summary, the relationship between biodiversity and productivity in the Inner Mongolia grassland may be driven by both the selection and complementarity effects in the grassland types with the lowest precipitation, but the selection effect may be the main mechanism in the grassland types with relatively high precipitation.

### 4.3 Community productivity is explained by different functional traits for different steppe zones

Plant functional traits are the result of long‐term evolutionary adaptations (Moles et al., 2009). As such, they are often regarded as indicators of ecosystem function (Fry et al., 2014; Liu et al., 2015). Functional traits can directly affect the flow of energy and material cycling and regulate ecosystem processes (Jiang, Zhang, & Wang, 2007). Many studies have demonstrated that plant functional traits can serve as a link between species and ecosystems (Cavanaugh et al., 2014; Liu et al., 2015). Therefore, investigating the relationship between plant functional traits and productivity is very important for understanding and improving ecosystem function.

In Inner Mongolia grasslands, plant height contributed 57.1% of the variation in community aboveground biomass and can thus be regarded as the key trait in explaining productivity (Table 3). Height allows plants to access more resources, including water, light, and essential nutrients, and therefore increase aboveground biomass (Falster, Brannstrom, Dieckmann, & Westoby, 2011; Westoby, Falster, Moles, Vesk, & Wright, 2002). Differences in climatic conditions result in the changing roles of functional traits, which is evidenced along the precipitation gradient in Inner Mongolia. While plant height is the key plant trait that explains community productivity, under the driest conditions of the *S. klemenzii* desert steppe, leaf area and specific leaf area become the most important (Table 3). With low (approximately 20%) cover in the drier steppe zones, plants do not need to compete for light resources but can instead achieve higher photosynthetic rates through increased leaf area (Vendramini et al., 2002). On the other hand, decreasing the specific leaf area minimizes the loss of water and leads to higher water use efficiency (Wilson, Thompson, & Hodgson, 2002). The strategy that plants generally follow to increase productivity under such dry conditions is to improve the water use efficiency of individual leaves by reducing their specific leaf area but at the same time increasing the total leaf area (more leaves) to enhance photosynthesis. For example, *Artemisia frigida*, a species common to the *S. klemenzii* desert steppe, is characterized by a small specific leaf area, an extremely high leaf density, and an overall large number of leaves. Competition for light resources becomes most important when community cover increases under more humid conditions, and plant height becomes the dominant trait explaining the aboveground biomass. From a management prospective, the improvement of grassland community productivity should target the preservation of species with high leaf area and low specific leaf area in the drier *S. klemenzii* desert steppe zone and favor taller plant species in the steppe zones with relatively higher precipitation.

## 5 Conclusions

Similar to other research conducted in natural plant communities, a positive relationship between diversity and community productivity was documented in the Inner Mongolia grassland. Our study also supported the previous conclusion that functional characteristics explain the variation in community aboveground biomass better than taxonomic diversity in the Inner Mongolia grassland. Furthermore, we found that functional dominance was the best predictor of community aboveground biomass. The selection effect may be the main mechanism explaining the relationship between biodiversity and community productivity. Furthermore, the selection effect and complementarity effect operate simultaneously in the grassland types with the lowest precipitation. Plant height is the most important functional trait promoting community productivity in the Inner Mongolia grassland. However, species with high leaf area and low specific leaf area are critical in improving community productivity in the grassland types with the lowest precipitation, such as the *S. klemenzii* desert steppe zones.

The Inner Mongolia grasslands are an important ecological barrier protecting North China and form the basis of animal husbandry in northern China. Research has shown that approximately 90% of the areas in the Inner Mongolia grasslands have experienced different degrees of degradation (Li, 1997). It is necessary to develop suitable approaches for restoring the grassland ecosystem and improving grassland function. The key component of this process should be to increase functional characteristics (especially some functional traits playing key roles in productivity) rather than species number. Based on our findings, species with high leaf area and low specific leaf area should be chosen for the relatively dry *S. klemenzii* desert steppe zones, and some species with taller heights should be screened for the relatively humid grassland types.

## Conflict of Interest

None declared.
